# Association of Pregestational Maternal Sleeping Disorders and Preeclampsia: A Retrospective Cohort Study and Review of the Literature

**DOI:** 10.7759/cureus.4338

**Published:** 2019-03-28

**Authors:** Nikolaos Georgiou, Zacharias Fasoulakis, Marianna Theodora, Vasileios A Pappas, Valentinos Papamanolis, Sofia Kalagasidou, Nikolaos Blontzos, Nikolaos J Kambas, Emmanuel N Kontomanolis

**Affiliations:** 1 Internal Medicine, East Kent University Hospitals NHS Trust, Kent, GBR; 2 Obstetrics and Gynecology, National and Kapodistrian University, Athens, GRC; 3 Mathematics, University of Ioannina, Ioannina, GRC; 4 Obstetrics and Gynecology, General Hospital of Korinth, Korinth, GRC; 5 Obstetrics and Gynecology, Bodosakio General Hospital of Ptolemaida, Ptolemaida, GRC; 6 Obstetrics and Gynecology, National and Kapodistrian University, Athens, USA; 7 Obstetrics and Gynecology, Democritus University of Thrace, University Hospital of Alexandroupolis, Alexandroupolis, GRC

**Keywords:** sleeping disorders, preeclampsia, pregnancy, rem sleep, adverse pregnancy outcomes

## Abstract

In this retrospective cohort study, primigravidas with normal pregnancies and women who developed preeclampsia (PE) were assigned to complete sleeping disorder questionnaires. The Crown-Rump length (CRL) of the first prenatal screening was used to determine the gestational age and the participants were assigned to complete the following questionnaires according to their everyday life before pregnancy: (1) Pittsburgh Sleep Quality Index (PSQI), (2) Epworth Sleepiness Scale (ESS), and (3) Athens Insomnia Scale (AIS). Women were also asked to evaluate their stress before pregnancy with the Beck Anxiety Inventory (BAI). The results of the women developing preeclampsia were analyzed to test the primary hypothesis that women with pre-existing to pregnancy sleep disorders are more likely to develop preeclampsia. Statistically significant differences were found between women who developed preeclampsia and women in the control group concerning sleeping disorder features before pregnancy of all three research tools, namely AIS (p<0.001), PSQI (p<0.001), and ESS (p=0.012<0.05). The results support that there is a possible statistical effect of pre-existing to pregnancy sleep disorders on the development of preeclampsia and women with pregestational sleep disorders request strict monitoring during pregnancy, however, further investigation with larger studies is needed to reach safe conclusions.

## Introduction

Preeclampsia (PE) constitutes a multisystemic disease comprising a leading cause of perinatal and maternal mortality and morbidity. At present, the diagnosis of this condition is carried out and based on non-specific clinical symptoms and laboratory tests. Numerous pathogenetic mechanisms have been accused of playing different roles in this disorder, among which an imbalance between angiogenic and antiangiogenic factors is believed to be the most important cause of the disease. On the other hand, pregnancy is correlated to several fluctuations in sleep quality and serious episodes of sleep disturbances. The authors of several studies have concluded that sleep disorders are frequent in pregnant women and the prevalence of such symptoms rises as pregnancy progresses [1]. Even though sleep disorders have been well-studied in pregnancy, there are no extensive reports objectively studying the connection of sleep disorders and preeclampsia. In this study, we aimed at evaluating the presence of sleep disorders prior to pregnancy as a risk factor for preeclampsia.

## Materials and methods

The study was conducted in the department of obstetrics and gynecology of the Democritus University of Thrace in cooperation with the department of obstetrics and gynecology of the General Hospital of Korinthos, during May 2017 to December 2017. We investigated the presence of sleep disorders as a risk factor for preeclampsia, by dividing women into two groups. Group A (control group) consisted of primigravidas attending the departments for monitoring of pregnancy, between 10 and 14 weeks of gestation, with problem-free pregnancies and pre-existing to pregnancy sleep disorders. The Crown-Rump length (CRL) of first-trimester prenatal screening was used to determine gestational age. The pregnant women were asked to complete the Pittsburgh Sleep Quality Index (PSQI), the Epworth Sleepiness Scale (ESS), the Athens Insomnia Scale (AIS), and the Beck Anxiety Inventory (BAI) as they experienced those sleep disorders before gestation. Women with a moderate to severe anxiety score were excluded from the study. The choice of healthy primigravidas and women without an anxiety score over 17 on the BAI questionnaire aimed at avoiding implications of PE and sleep disorders to other variables, such as anxiety, health problems, and previous adverse perinatal outcomes. Due to the low frequency of the disease and the random import of patients with PE (29 patients with PE that presented to our clinics) during the same period, women with PE that attended the clinics formed the second group (Group B), completing the questionnaires even though their pregnancies were beyond the 14th week of gestation. Group A consisted of forty-nine (49) primigravidas with normal ongoing pregnancies that agreed on participating in the study and Group B consisted of twenty-two women (22) with preeclampsia. Five out of 29 patients (17,3%) supported that sleeping disorders did not occur before pregnancy but started right after conception, one patient reported severe anxiety while one woman refused to participate. As a result, all women who did not meet criteria (five patients with a sleep disorder that did not exist before pregnancy, one patient with severe anxiety, and one patient that refused participation) were excluded from the study while all the other patients (71) agreed on participating in this study.

Statistical analyses were performed using Statistical Package for Social Sciences (IBM SPSS, Armonk, NY, US) version 22.0. When the appropriate assumptions were met, the parametric t-test was used in order to check the comparison of the means of the two study groups (age). In cases that the equal variance assumption was not met, the Welch test was used, body mass index (BMI), and in those that data was not normally distributed, the determination of significant differences between the medians of the two groups was carried out using the non-parametric unpaired Mann-Whitney U test (AIS, PSQI, ESS). Comparisons of proportions of categorical variables between the two groups were applied with Pearson's Chi-squared test (PSQI, ESS) and the equivalent Fisher's exact test (AIS) when appropriate or the numerical assumptions of the observed frequencies were not met. On all statistical tests, the significance level was set at 0.05.

## Results

With regards to the two quantitative characteristics of the participating women, namely, age and BMI, there were no significant differences regarding statistics between the two groups, p-value=0.359>0.05 and p-value=0.071>0.05 for age and BMI, respectively. This isolates any influence that these variables could have on the qualitative characteristics regarding sleep disorders that are measured through the three questionnaires. The mean age of the participating women, regardless of group, was 29.6 years and the mean BMI was 21.64.

The statistical analysis of the research data of the three questionnaires revealed the existence of statistically significant differences in all performed tests. In particular, in all indexes, AIS (p-value <0.001), PSQI (p-value <0.001) and ESS (p-value = 0.012 <0.05), there were statistically significant differences between the two groups. The control group scored apparently lower values than the preeclampsia group. This result is reinforced by the fact that in all indexes, AIS (p-value=0.017<0.05), PSQI (p-value=0.005<0.05) and ESS (p-value=0.025<0.05), statistically significant differences in the distribution of the response frequencies of women in both groups were identified. Women with preeclampsia appear to have higher rates of insomnia (OR = 5.03), less sleep quality (OR = 4.45), and more sleepiness (OR = 3.27) before pregnancy, compared to women in the control group. This finding may indicate a possible individual or joint influence of the above sleep disorders on the occurrence of preeclampsia during pregnancy (Table 1). 

**Table 1:** Maternal data and questionnaire results between preeclampsia and the control group. No significant differences regarding age and BMI were observed between the two groups. The control group recorded smaller medians in AIS, PSQI (p-value <0.001) and ESS (p-value 0.012) questionnaires while preeclampsia group reported higher rates of insomnia (OR = 5.03), less sleep quality (OR = 4.45), more sleepiness (OR = 3.27) and increased sleep difficulties compared to control group, according to AIS (p-value=0.017<0.05), PSQI (p-value=0.005<0.05) and ESS (p-value=0.025<0.05) results. BMI: body mass index; AIS: Athens Insomnia Scale; PSQI: Pittsburgh Sleep Quality Index; ESS: Epworth Sleepiness Scale

	Group A: Control Group (Total: 49)	Group B: Preeclampsia Group (Total: 22)	p-value
Age (years; mean ± SD)	30.20 ± 6.27	28.27 ± 8.78	0.359 (Welch)
ΒΜΙ (Kg/m^2^; mean ± SD)	21.98 ± 2.31	20.87 ± 2.45	0.071 (T-test)
AIS (median, 25^th^ – 75^th^ percentile)	0 (0 – 4)	5 (4 – 7)	<0.001 (Mann-Whitney)
AIS (no, %)	-	-	0.017 (Fisher)
≥ 6	5 (10.2)	8 (36.4)	0.017 (Fisher)
< 6	44 (89.9)	14 (63.6)	0.017 (Fisher)
OR (95% CI)	5.03 (1.41 - 17.89)	5.03 (1.41 - 17.89)	5.03 (1.41 - 17.89)
PSQI (median, 25^th^ – 75^th^ percentile)	1 (0 – 4.5)	6 (3.75 – 12.25)	<0.001 (Mann-Whitney)
PSQI (no, %)	-	-	0.005 (Chi-squared)
≥ 5	12 (24.5)	13 (59.1)	0.005 (Chi-squared)
< 5	37 (75.5)	9 (40.9)	0.005 (Chi-squared)
OR (95% CI)	4.45 (1.53 – 12.99)	4.45 (1.53 – 12.99)	4.45 (1.53 – 12.99)
ESS (median, 25^th^ – 75^th^ percentile)	4 (0 – 8)	6 (4 – 10.25)	0.012 (Mann-Whitney)
ESS (no, %)	-	-	0.023 (Chi-squared)
≥ 6	15 (30.6)	13 (59.1)	0.023 (Chi-squared)
< 6	34 (69.4)	9 (40.9)	0.023 (Chi-squared)
OR (95% CI)	3.27 (1.15 – 9.31)	3.27 (1.15 – 9.31)	3.27 (1.15 – 9.31)

## Discussion

The term "sleep" is characterized by transitions between three unique states: rapid eye movement (REM) sleep, which is correlated to dreaming, non-rapid eye movement (N-REM) sleep, and the stage of wakefulness. During this state, there are specific relations between the electrical activities of the brain, as recorded by an electroencephalogram. Although the transmission of nerve impulses in the brain during REM sleep is functioning at approximately the same extent as during wakefulness, temporary muscle paralysis takes place throughout the phase of REM sleep. Difficulties in sleeping occur in all the different stages of sleeping mentioned above [2].

There are numerous sleep disorders that patients have to deal with, and because of them, they have to face harmful effects on their lifestyle.

Insomnia constitutes the most common sleep obstacle, which happens when patients have trouble falling or staying asleep or do not feel revived in the morning. Two significant symptoms of insomnia can be described as problems in falling asleep and waking up too early in the morning. It causes adverse impacts on patients’ well-being, leading to poor overall quality of life. Surveys conducted in the US have shown that more than half the American population experiences symptoms of insomnia a few times per week, whereas almost one-fifth of those have to deal with chronic insomnia, which affects their daytime functioning [3-4].

The term “hypersomnias’’ refers to conditions in which patients feel excessively sleepy during the whole day. They may also have other sleep-related difficulties accompanied by a lack of energy and trouble thinking clearly. Narcolepsy is the most dominant hypersomnia [5].

Parasomnias are a group of sleep diseases that entail unpredictable experiences, which occur while the patients are falling asleep, sleeping, or waking up. Parasomnias may include abnormal movements, behaviors, emotions, and perceptions or dreams such as sleepwalking [6].

In addition, there is another category, which entails ailments that can cause a sleeping deficiency and is divided into two sub-sections. These include sleep disorders that are caused by breathing difficulties (such as sleep apnea) and those that are based on malfunctions in the circadian rhythm (such as jet lag) [7-9].

Preeclampsia is a frequent syndrome related to pregnancy, which is mainly described by de novo high blood pressure and proteinuria (≥300 mg/day or a spot urine protein/creatinine ratio ≥30 mg/mmol) after 20 weeks of gestation while in the absence of proteinuria, preeclampsia is diagnosed as hypertension in association with renal insufficiency (creatinine ≥1.1 mg/dL or oliguria), raised transaminases with possible severe right upper quadrant or epigastric pain, haematological disturbances (platelet count <100.000/μL), and pulmonary edema or new-onset cerebral or visual disturbances. Surveys on pregnant women have shown that almost 2%-8% of all pregnancies are affected by this disease, and it accounts for one quarter of all maternal deaths and perinatal morbidity and mortality [10].

Although preeclampsia is something different than simple gestational hypertension in combination with proteins detected in pregnant women’s urine samples, the development of proteinuria is still one significant and objective diagnostic measure of this disorder [1,11].

Surprisingly, a placenta, but not the fetus, is required for the development of preeclampsia, as preeclampsia can be triggered in patients carrying hydatidiform moles. As a result, the most effective treatment for pre-eclampsia is the delivery of the placenta. The traditional view of the pathogenetic mechanisms involved in pre-eclampsia is that an ischaemic placenta produces toxins that, after being released into the maternal circulation, cause the clinical symptoms of preeclampsia. These soluble factors are thought to be responsible for intravascular inflammation, endothelial cell dysfunction, and the activation of the hemostatic system; accordingly, preeclampsia is considered to be primarily a vascular disorder. Besides, a variety of organs are included in the manifestations of preeclampsia such as the kidneys, liver, brain, heart, lung, pancreas, and the vasculature [1,12].

Precipitating factors that cause preeclampsia are diabetes, chronic hypertension before pregnancy, multiple pregnancies, chronic kidney disease, family history of preeclampsia or eclampsia, immune disorders and obesity [11-12].

Previous episodes of preeclampsia do not necessarily predict the occurrence of this disease in subsequent potential pregnancies. However, when the first episode of preeclampsia occurs in a pregnant patient, the chances of recurrence in the next pregnancies are higher [13].

Preeclampsia is described to happen in two different stages. In stage one, there is a distinct decrease in placental perfusion. The reduced blood flow through the placenta is proposed to produce substances that, in the maternal environment, can initiate the ensuing multisystem abnormalities (stage two). The endothelial dysfunction is part of a generalized intravascular inflammatory reaction involving leukocytes and the clotting and complement systems. It seems that poor placental blood flow is not the main cause of preeclampsia, but it is a dominant predisposing factor. Studies also support that preeclampsia could also be induced by low serum levels of vitamin D. More specifically, 1,25-dihydroxyvitamin D3 - the active form of vitamin D - has been suggested to participate in the transcription and function of the genes associated with placental physiology. The immunomodulatory properties of 1,25-dihydroxyvitamin D are related to normal immune modulation and vascular function, while abnormal implantation, inflammatory reactions, and hypertension are being connected to vitamin D deficiency [14].

As mentioned before, preeclampsia constitutes a multisystemic disease comprising a leading cause of perinatal and maternal mortality and morbidity. At present, the diagnosis of this condition is carried out and based on non-specific clinical symptoms and laboratory tests. Numerous pathogenetic mechanisms have been blamed for playing different roles in this disorder; among them, an imbalance between angiogenic and antiangiogenic factors are believed to be the most important cause of the disease. Thus, the diagnosis and sub-classification of preeclampsia based on biomarkers of specific aetiologies might be useful for identifying patients at risk, monitoring disease progression, and providing effective interventions. Further investigation on the mechanisms of abnormal placentation could help in acquiring extra knowledge of the pathogenesis of preeclampsia as well as other disorders, such as certain types of spontaneous preterm delivery and potential restrictions in the growth of the fetus.

Pregnancy is correlated to several fluctuations in sleep quality and serious episodes of sleep disturbances. The authors of several studies have come to the conclusion that sleep disorders are frequent in pregnant women and the prevalence of such symptoms rise as pregnancy progresses [1]. Studies that have been carried out among pregnant women have proven that sleep disturbances during the first two trimesters may result in disruptions of placental and fetal development, leading to pregnancy losses [15]. Also, fluctuations in sleep patterns during pregnancy can lead to the increased secretion of blood hormones (such as cortisol) and abnormalities in insulin and glucose metabolism [15]. Although sleeping difficulties have been studied in pregnancy, there are no extensive studies objectively evaluating the prevalence of sleep disorders in pregnancy.

On the other hand, sleeping difficulties have become an important public health issue and sleep disorders, such as insufficient sleep duration and poor sleep quality, have become common during pregnancy. Pregnant women are easily affected by sleep disruption, deprivation, and sleep disorders. Inadequate sleep duration and poor sleep quality during pregnancy may increase the risk of adverse pregnancy outcomes, including growth restriction of the fetus and postpartum depression [1,15].

Insufficient sleep is associated with a variety of adverse health behaviors while poor sleep quality and significant fluctuations in the duration of sleep during pregnancy are associated with an increased risk of health problems, such as gestational diabetes [16].

Since there is not enough evidence investigating the relationship of poor sleep quality in the pregestational period with adverse perinatal outcomes, the frequent reference to sleep problems by pregnant women triggered our research. Almost all of the patients complained about sleeping difficulties during pregnancy, however, many of the pregnant women mentioned sleeping disorders that pre-existed for many years. One of these patients was suffering from preeclampsia. This case led our investigation on searching for a connection between those two conditions.

It is not clear whether stress was the most important factor in PE development on the population we studied, although it certainly plays a significant role. Moreover, due to the general economic instability, we did not take into account the socio-economic status of each of the patients, which would also directly contribute as a variable to the anxiety and general sleeping conditions of our sample, with our results limited to the specific criteria mentioned. Thus, in this study, we decided to choose only primigravidas without a history of health problems or excessive anxiety, in order to clear other variables from our research profile. Even though the participants were asked to complete the BAI questionnaire, we tried to avoid correlating clinical anxiety with sleep disorders because we could not ensure if anxiety was definitely a predominant feature, or if it was due to pregnancy, or if it was derived from the parental role that the parents were going to take. Even though our findings indicated a possible individual or joint influence of sleep disorders on the occurrence of preeclampsia during pregnancy, larger studies are needed in order to reach safe conclusions.

## Conclusions

Maternal sleep plays a vital role in fetal well-being because the proteins that flow through the blood, especially growth hormones, peak during sleep. Thus, maternal health and adequate fetal development require good quality of sleeping during pregnancy. Although at present, there is no significant number of studies that directly link sleep disturbances with severe pregnancy outcomes, researchers have found that loss of sleep may cause detrimental effects on both the fetus and the mother such as maternal comorbidities, emergency cesarian deliveries, and increased risk pregnancies. Our results reveal a possible connection of pregestational sleeping disorders to preeclampsia, however, further investigation needs to be carried out in order to clarify whether sleep disorders are indeed connected to the disease and if they actually portend or contribute to PE.
